# ExplainTS: a benchmark dataset of pretrained models and *post-hoc* explanations for time-series classification

**DOI:** 10.3389/frai.2026.1759110

**Published:** 2026-05-05

**Authors:** Maciej Mozolewski, Szymon Bobek, Grzegorz J. Nalepa

**Affiliations:** 1 Jagiellonian Human-Centered AI Lab, Mark Kac Center for Complex Systems Research, Jagiellonian University, Kraków, Poland; 2 Department of Human-Centered Artificial Intelligence, Institute of Applied Computer Science, Faculty of Physics, Astronomy and Applied Computer Science, Jagiellonian University, Kraków, Poland

**Keywords:** time-series classification, explainable AI, *post-hoc* explanations, SHAP, LIME, Anchor, *Post-hoc* Attribution Rules (PHAR), benchmark dataset

## 1 Introduction

Reproducibility in machine learning (ML) traditionally relies on shared datasets and fixed train–test splits. However, evaluating Explainable AI (XAI) introduces additional complexity, as explanations depend on both the data and the specific underlying model. In time-series classification, where complex black-box neural networks are increasingly deployed (Fawaz et al., 2020), the lack of standardized XAI benchmarks (Höllig et al., 2023) severely hinders progress. Currently, most studies retrain models and recompute explanations from scratch. This not only inflates computational costs but also makes it nearly impossible to disentangle genuine XAI methodological improvements from model variation, preprocessing differences, or the inherent run-to-run variance of perturbation-based *post-hoc* explainers (Ribeiro et al., 2018).

To ensure reproducibility, we extend standard ML practices by treating datasets, trained models, and explanation pipelines as fixed benchmark artifacts. While mature frameworks like *aeon* (Middlehurst et al., 2024) standardize predictive evaluation, and dedicated XAI libraries enable the calculation of explanations, they do not distribute precomputed, resource-intensive XAI outputs. Providing this frozen baseline layer eliminates the computational overhead of recomputing ad hoc explanations, enabling consistent XAI evaluation.

Beyond reproducibility, generating sampling-based explanations for time-series is often computationally more expensive than training the models. While traditional ML repositories like *OpenML* and the *UCR Time Series Classification Archive* successfully reduce redundant computation, no equivalent initiative stores precomputed *post-hoc* explanations alongside underlying models for diverse time-series tasks. Existing XAI frameworks, such as *Captum* (Kokhlikyan et al., 2020), *Quantus* (Hedström et al., 2023), and *Alibi* (Klaise et al., 2021), offer valuable tooling but still require resource-intensive model retraining and explanation recomputation.

Furthermore, while XAI evaluation datasets exist (Tritscher et al., 2020), particularly in vision and NLP (Arras et al., 2022; DeYoung et al., 2020; Yalcin et al., 2021; Liu et al., 2021; Zhang et al., 2025), they focus on ground-truth annotations rather than reproducible *model plus explanation* pipelines. Similarly, time-series benchmarks like *Exathlon* (Jacob et al., 2021) and *XTSC-Bench* (Höllig et al., 2023) do not distribute precomputed *post-hoc* explanations. Unlike standalone software libraries, *ExplainTS* acts as a complementary data resource. By providing frozen, ready-to-use explanation artifacts it allows researchers to evaluate new XAI methods and metrics without the massive computational burden of regenerating baselines.

Our main contribution is *ExplainTS*, a comprehensive repository comprising 103 diverse classification tasks. Its core features include:

- Shared datasets, frozen train–test splits, and pretrained *ConvLSTM-based* baselines for consistent evaluation of XAI methods.
- Precomputed outputs from multiple model-agnostic explainers [*SHAP* (Lundberg and Lee, 2017), *LIME* (Ribeiro et al., 2016), *Anchor* (Ribeiro et al., 2018), and *PHAR* (Mozolewski et al., 2026)], significantly reducing the computational cost of experiments and eliminating run-to-run stochastic variance.
- A stable artifact layer (data, models, and explanations) for evaluating advanced explanation-quality metrics (e.g., Lipschitz quotient or Jaccard Index) without re-running expensive pipelines.
- An educational case study and interactive templates demonstrating how to utilize these precomputed artifacts for downstream XAI auditing, lowering the entry barrier for students and researchers.

**Figure 1:**
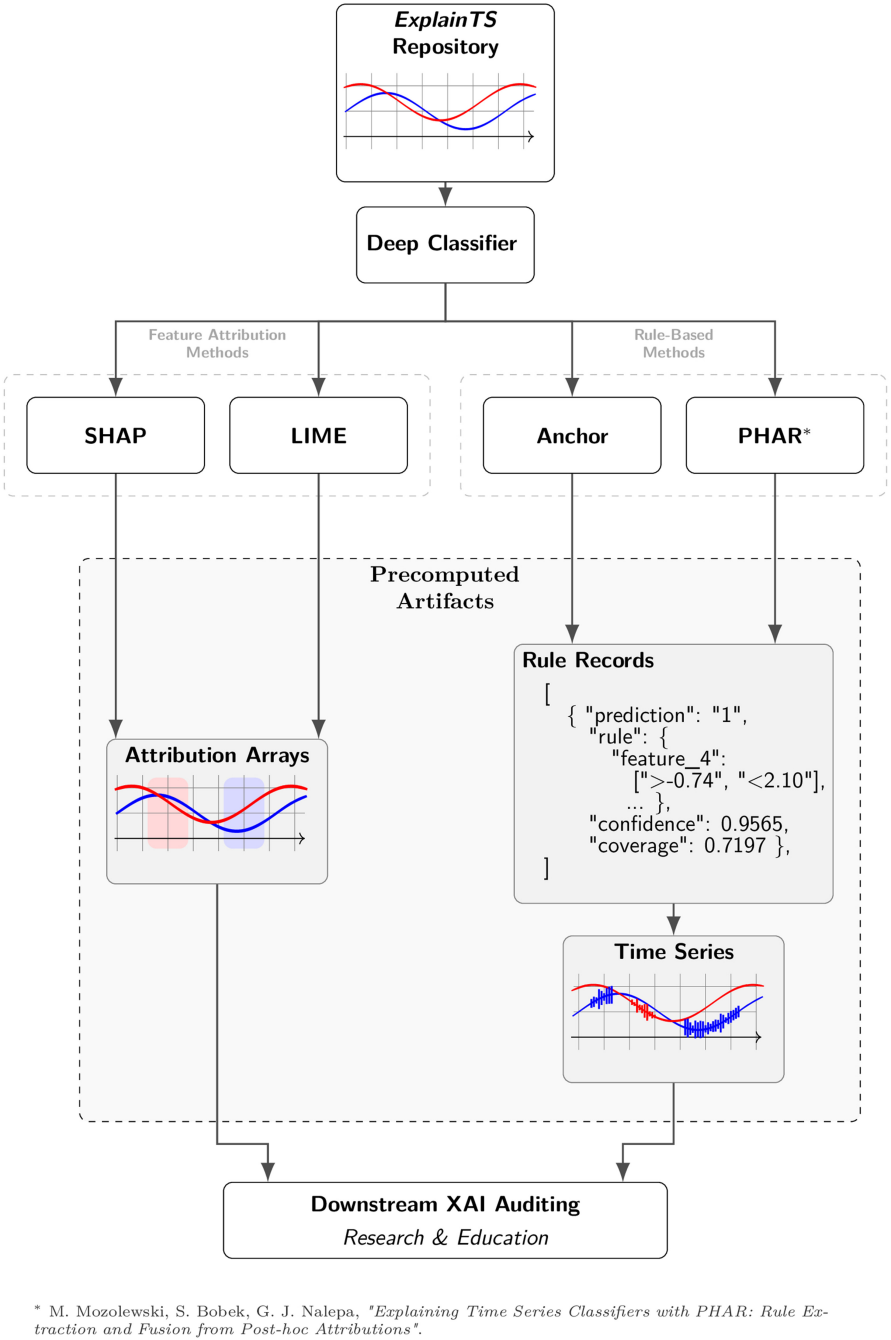
Repository overview: shared datasets in a common format, a pretrained deep classifier, and reference *post-hoc* explanations (SHAP, LIME, Anchor, PHAR). Source: Mozolewski et al. (2026).

The repository structure (Figure 1) is fully extensible via open-source Python code (github.com/mozo64/papers/tree/main/zenodo-ucr), allowing researchers to seamlessly integrate their own custom metrics or algorithms without re-running expensive baseline pipelines. We actively encourage the research community to contribute novel explainers and evaluation protocols via pull requests, paving the way for collaborative, iterative updates to the Zenodo benchmark.

The remainder of this article is organized as follows. Sections 2 and 3 detail the benchmark construction, data characteristics, and intended uses, while Section 3.4 outlines the Zenodo archive structure. Importantly, comprehensive dataset statistics, exact model performance metrics, and detailed repository layouts are provided exclusively in Supplementary Tables S1–S4.

## 2 Methods

### 2.1 Datasets and preprocessing

The benchmark covers 103 tasks from the *UCR/UEA* time-series archives (Dau et al., 2019; Bagnall et al., 2018), including 83 univariate and 20 multivariate datasets. We excluded datasets where the standard ConvLSTM architecture failed to converge effectively, ensuring the repository contains only reliable models for subsequent explanation analysis. The selected datasets vary significantly in scale and complexity: total instance counts range from 30 to 24,000 (median: 553), time-series lengths span from 8 to 1,751 time steps (median: 235), and the number of target classes varies between 2 and 60 (median: 3). While univariate datasets contain a single channel, the multivariate tasks include up to 144 dimensions. Detailed descriptive statistics for each individual dataset are provided in Supplementary Tables S1–S3. Some multivariate tasks (e.g., *Libras, PenDigits*) originate from the *UCI* Machine Learning Repository, but here we rely on the curated UCR/UEA variants of these datasets.^1^ No new measurements were collected, and the original licensing terms for all third-party datasets remain with their owners.

The raw time-series were downloaded programmatically using the eon loader (Middlehurst et al., 2024). Each channel was scaled to zero mean and unit variance with the scikit-learn StandardScaler (Pedregosa et al., 2011) fitted to the training split and reused in the test split. Each series of length *T* was segmented into blocks of shape (*n*_steps_, *n*_length_, *F*), where *n*_steps_ is the third-smallest divisor of *T* above 2 and *F* is the number of channels. This segmentation yields tensors with a consistent shape, so that the same convolutional architecture can be instantiated and trained separately on each data set, and converts each multichannel time-series into a (steps × length × channels) representation compatible with the ConvLSTM-based implementation. While this dynamic heuristic avoids per-dataset hyperparameter tuning and proved robust across the majority of tasks, it represents a compromise. We did not perform a granular sensitivity analysis for each dataset to quantify potential temporal distortions; however, maintaining a unified architecture was prioritized to establish a stable baseline for XAI evaluation rather than maximizing predictive performance through custom preprocessing. All datasets use a predefined stratified 75/25 train–test split that is reused across all experiments. The accuracy of ConvLSTM-based classifier and the availability status of the SHAP, LIME, Anchor and PHAR explanations for all multivariate and univariate datasets are reported in Supplementary Tables S1–S3.

### 2.2 Classifier architecture and training

**Figure 2:**
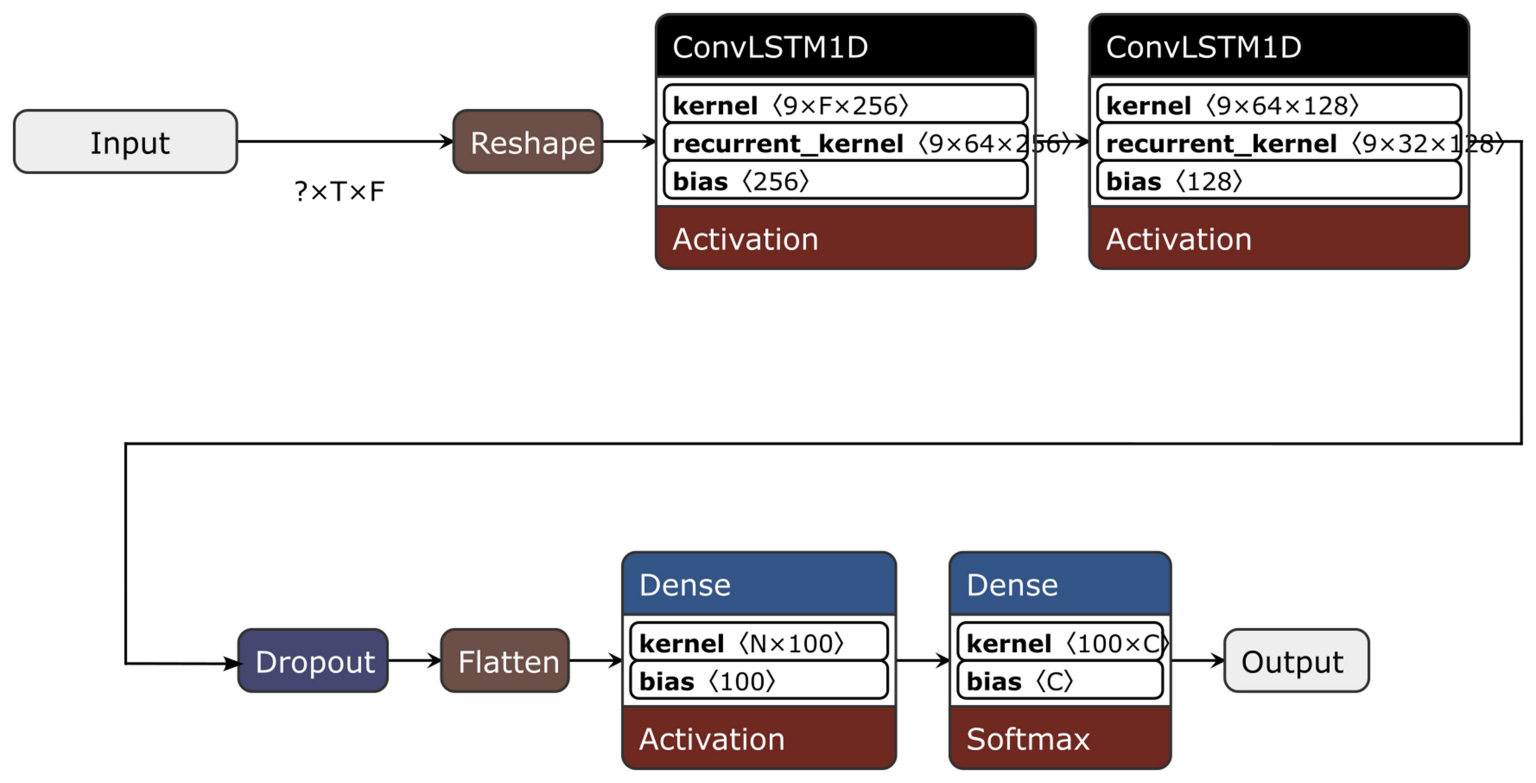
Overview of the ConvLSTM-based classifier used in ExplainTS, implemented with ConvLSTM1D layers on reshaped inputs, concluding with a Softmax output. Variables denote the original sequence length (*T*), number of features/channels (*F*), flattened representation size (*N*), and number of classes (*C*).

We employ a unified convolutional recurrent classifier architecture across all datasets. As illustrated in Figure 2, the model leverages stacked *ConvLSTM1D* layers applied to reshaped inputs to process time-series data.

The network consists of two such layers with 64 and 32 filters (kernel size 9, *ReLU* activations), followed by dropout (rate 0.5), flattening to a consistent-length representation *N*, a dense layer with 100 *ReLU* units, and a softmax output layer over the set of *C* target classes. The class imbalance is handled with class weights derived from the training labels. The models are trained with *Adam* (Kingma and Ba, 2015) and categorical cross-entropy for 25 epochs using a batch size of 64. The same architecture and training protocol are applied across all tasks without per-dataset hyperparameter tuning, so that the model side remains constant and the analysis can focus on the behavior of *post-hoc* explainers.^2^

While state-of-the-art models like ROCKET Dempster et al. (2020) and HIVE-COTE 2.0 Middlehurst et al. (2021) excel in accuracy, their complex ensemble structures make perturbation-based XAI computationally prohibitive. Even with our standard ConvLSTM baseline, computing explanations using methods like DeepSHAP remains highly resource-intensive. Crucially, this fully differentiable architecture not only represents a typical deep learning black-box but also ensures future compatibility with gradient-based explainers.

### 2.3 Explanation generation pipeline

For each trained *ConvLSTM-based* classifier, we generate *post-hoc* explanations using four model-agnostic methods: *SHAP* (Lundberg and Lee, 2017) for additive feature attributions, *LIME* (Ribeiro et al., 2016) for local surrogate models, *Anchor* (Ribeiro et al., 2018) for baseline rule-based explanations, and our recently proposed *PHAR* framework (Mozolewski et al., 2026) for high-coverage interval rules. These methods provide attribution-based and rule-based views of model decisions.

To provide the community with standard, highly reproducible out-of-the-box baselines, SHAP, LIME, and Anchor are deliberately executed without dataset-specific tuning.

*DeepSHAP* is executed using the reference Python implementation shap.DeepExplainer, utilizing a background distribution of 1,000 random samples drawn from the training set. *LIME* uses LimeTabularExplainer on flattened (*T*×*C*) feature vectors (where *T* is the number of time steps and *C* is the number of channels), configured with the default 5,000 perturbation samples and continuous feature discretization (discretize_continuous=True) to ensure extraction stability.^3^

*Anchor* targets a 0.95 precision threshold with quartile discretization, using a grid-search heuristic to maximize the product of coverage, precision, and condition count. To handle continuous signals, it employs a multi-hour cascading retry mechanism, falling back from the alibi package to anchor-exp.^4^ Because Anchor frequently fails to extract valid rules despite this massive computational budget, we integrated PHAR to guarantee stable, high-coverage rule generation across the benchmark.

The PHAR pipeline inherently relies on a two-stage hyperparameter optimization (HPO) process to extract robust rules.^5^ First, a multi-objective Pareto-front search (implemented via the optuna hyperparameter optimization framework) explores the optimal configuration (base explainer: SHAP or LIME, threshold percentiles, and perturbation variance) on a small, stratified sample of the test set, maximizing a trade-off between rule confidence and coverage while minimizing sparsity. Second, the single best configuration is applied globally to extract robust interval rules for both the full training and test splits. Consequently, alongside the final rules (formatted consistently with Anchor records), the repository provides comprehensive metadata detailing the winning parameters (phar_metadata.json) and the complete HPO trial history (phar_trials_log.jsonl), ensuring full reproducibility of the extraction process.

SHAP and LIME explanations are stored as value arrays, while Anchor and PHAR explanations are stored as JSON-like rule records with metadata fields such as *confidence* and *coverage* of each individual rule. The listing 1 shows an example of an Anchor/PHAR rule record, where the conjunction specifies the constraints at the feature-level, the *confidence* is the empirical precision of the rule and the *coverage* is the fraction of the data set that satisfies it.

Listing 1 **Example Anchor rule record used in ExplainTS (PHAR folows the same schema).**


[
   {
      "index": 123,
      "success": true,
      "prediction": "1",
      "rule": {
        "feature_1": [">-0.74", "<=-0.12"],
        "feature_11": [">3.94", "<=4.01"],
        "feature_110": [">1.89", "<=2.91"]
      },
      "confidence": 0.9565,
      "coverage": 0.7197
   }
]

All training and explanation computations were run on two NVIDIA RTX A5500 GPUs. The same explanation pipeline is applied across all datasets, which makes comparisons between explainers fair and repeatable while still allowing users to rerun the methods with alternative settings (e.g., different background sets or sampling budgets) on top of the predefined splits and pretrained models in the benchmark dataset.

### 2.4 Code repository

All notebooks and helper scripts used to build the ExplainTS benchmark dataset are hosted on a public GitHub repository.^6^ The main top-level directories are:

***notebooks/*** ExplainTS_CaseStudy.ipynb (an educational case study calculating XAI stability metrics), UCR-train.ipynb (training ConvLSTM-based models), UCR-explainers-lime-shap.ipnb, UCR-explainers-anchor.ipynb, and UCR-explainers-phar.ipynb (running SHAP/LIME, Anchor, and PHAR explainers), and datasets_summary.ipynb (extracting dataset statistics and calculating model accuracies).
***services/*** model_manager.py, model_server.py, and simple job-listing utilities used to parallelise explanation jobs for *Anchor*.
***results/*** contains data_and_model_evaluations.csv with properties of the datasets, train/test splits and accuracies generated by the datasets_summary.ipynb notebook.
***scripts/*** bash utilities for dataset assembly, including compress_models.sh and compress_explainers.sh (packaging models and individual artifacts), filter_move.sh (validating completeness and organizing bundles), compress_all.sh (creating the final Zenodo archives), and report.sh (generating coverage statistics).

These resources facilitate full reproducibility and provide templates for integrating new explainers. Detailed Zenodo archive structures and their exact contents are documented in Supplementary material Section 1.2 and Supplementary Table S4.

## 3 Benchmark characteristics and usage notes

We characterize the ExplainTS benchmark along two axes: the performance of a shared *ConvLSTM-based classifier* baseline across datasets and the availability of *post-hoc* explanations (*DeepSHAP, LIME, Anchor*) on the train and test splits.

### 3.1 Model performance and explanation availability

While SHAP achieves full coverage and LIME covers all except *FaceDetection*, Anchor rules (exported with *confidence* and *coverage* metadata) successfully span only four of 20 multivariate and 26 of 83 univariate datasets. To overcome Anchor's inherent difficulty with continuous time-series, we integrated the rule-based PHAR framework, which guarantees robust extraction and restores full coverage across all 103 tasks.

Although our vanilla ConvLSTM baseline generally underperforms top UCR/UEA leaderboards, these models remain highly valuable for XAI: their explanations faithfully expose internal logic, allowing researchers to analyze structural flaws and debug misclassifications.

Dataset-specific metrics are detailed in Supplementary Tables S1–S3.

### 3.2 Educational demonstration and usage tutorial

To practically demonstrate the utility of the benchmark, we provide an educational template, ExplainTS_CaseStudy.ipynb. Importantly, this script serves strictly as a technical proof-of-concept and a usage tutorial, not as a comprehensive empirical evaluation of XAI methods. It illustrates a plug-and-play workflow where users can seamlessly load a pretrained time-series classifier alongside its test data to verify baseline classification accuracy. Subsequently, it shows how to fetch the corresponding precomputed explanation artifacts, covering both continuous feature attributions (*shap.zip, lime.zip*) and discrete, logic-based rules (*anchor.zip, phar.zip*). Furthermore, the notebook demonstrates how to visualize these explanations directly over the raw time-series signals, including helper routines for exporting publication-ready PDF figures, and provides example implementations of quantitative XAI stability metrics, such as the Jaccard Index for rule agreement and the local Lipschitz quotient for attribution robustness. By completely bypassing the computationally expensive phases of model training and explainer optimization, this template highlights how researchers can use the repository to immediately focus on prototyping custom metrics and advancing XAI evaluation.

### 3.3 Intended and typical uses of the ExplainTS benchmark

Since pretrained models and *post-hoc* explanations are precomputed for all tasks, the primary use of the benchmark is to facilitate the evaluation of new explainer variants, explanation-quality metrics, or visualization techniques without the overhead of repeating model training or explanation sampling. The diversity of the 103 datasets further enables large-scale meta-analyses, allowing researchers to investigate how data characteristics correlate with explanation properties. Users can, for example, derive custom stability indicators for SHAP and LIME, filter Anchor and PHAR rules by *confidence* and *coverage*, or conduct direct cross-method comparisons (e.g., contrasting attribution maps with discrete logic rules). By freezing the data splits and model weights, *ExplainTS* ensures that all such comparative experiments are strictly isolated from the underlying model and run-to-run variance.

Furthermore, ExplainTS provides an accessible entry point for teaching and rapid prototyping. By combining real-world datasets with a unified architecture and a public code repository (Section 2.4), it significantly lowers the barrier to entry for applied XAI on time-series.

Users can rely on the provided educational notebooks, such as the demonstration discussed in the previous section, as practical templates to load artifacts, experiment with visualizations, and safely extend the benchmark with custom metrics or explainer implementations. Ultimately, *ExplainTS* is designed as a living, community-driven resource; we strongly invite researchers to submit their locally computed explanation artifacts to be incorporated into subsequent, jointly-authored releases of the Zenodo record.

### 3.4 Limitations and future extensions of the benchmark

As a uniform benchmark, *ExplainTS* has several inherent limitations. Currently, precomputed artifacts are restricted to four model-agnostic methods (*DeepSHAP, LIME, Anchor, PHAR*). Although PHAR mitigates Anchor's coverage gaps, extracting discrete rules from continuous time-series remains challenging; users must carefully account for the exported *confidence* and *coverage* metadata. Furthermore, gradient-based (e.g., *Integrated Gradients, Grad-CAM*) and counterfactual explainers are not yet included. To address this, we actively invite the research community to contribute new explainers to the code repository, with the goal of incorporating community-generated artifacts into future updates of the Zenodo record.

Second, lacking ground-truth relevance masks for real-world UCR/UEA data, evaluating explanation faithfulness requires proxy metrics. Consequently, SHAP and LIME outputs must be interpreted as perturbation-based approximations, not causal truths. Structurally, the current benchmark is limited to classification tasks using a single ConvLSTM baseline. Future extensions may include alternative architectures (e.g., *InceptionTime, Transformers*), forecasting tasks, and reference implementations of cross-dataset XAI metrics.

Finally, the UCR/UEA archive exhibits domain imbalance (heavily dominated by sensor, motion, and image data), potentially limiting XAI generalizability. Ethically, while *ExplainTS* lacks sensitive data, deploying such *post-hoc* pipelines on real-world sequences (e.g., health records) requires strict safeguards to prevent instance-level feature leakage via local explanations.

## Data Availability

The ExplainTS artifacts are available at Zenodo (10.5281/zenodo.15173400). The deposit includes: train test.zip (NumPy arrays, 75/25 split; original UCR/UEA licenses apply); models.zip (TensorFlow SavedModels and .h5 files for loading flexibility); and explanation archives: shap.zip and lime.zip (NumPy arrays), anchor.zip (JSON rule sets with metadata), and phar.zip (JSON rules, plus .jsonl hyperoptimization histories and final best .json results).

## Footnotes

1 UCR/UEA archive: https://www.cs.ucr.edu/~eamonn/time_series_data_2018/ and https://www.timeseriesclassification.com/; selected datasets (e.g., *PenDigits*) originate from UCI: https://archive.ics.uci.edu/.

2 The training pipeline is available at: https://github.com/mozo64/papers/blob/main/zenodo-ucr/notebooks/UCR-train.ipynb

3 Reference implementations: github.com/shap/shap, github.com/marcotcr/lime. SHAP and LIME extraction: https://github.com/mozo64/papers/blob/main/zenodo-ucr/notebooks/UCR-explainers-lime-shap.ipynb

4 Reference implementations: github.com/marcotcr/anchor, github.com/SeldonIO/alibi. Anchor extraction: https://github.com/mozo64/papers/blob/main/zenodo-ucr/notebooks/UCR-explainers-anchor.ipynb

5 PHAR extraction: https://github.com/mozo64/papers/blob/main/zenodo-ucr/notebooks/UCR-explainers-phar.ipynb

6 https://github.com/mozo64/papers/tree/main/zenodo-ucr.
