# Supplementary material for: ExplainTS: a benchmark dataset of pretrained models and post-hoc explanations for time-series classification
Source: Front Artif Intell. 2026 May 5;9:1759110. doi: 10.3389/frai.2026.1759110 (PMC13183791; doi:10.3389/frai.2026.1759110)
Supplement: Supplementary file 1 [file Data_sheet_1.pdf]

Supplementary Material

1 SUPPLEMENTARY TABLES FOR THE EXPLAINTS BENCHMARK DATASET

Supplementary Tables S1–S3 detail the characteristics of the *ExplainTS* benchmark datasets, including train/test split sizes (Train, Test), sequence length (Len.), dimensionality (Dim.), and the number of classes (Classes). They also report the test-set accuracy of the unified *ConvLSTM-based classifier* (Acc. (%)) alongside the successful generation of local explanations for both train and test set: LIME, SHAP, Anchor, and PHAR[1]. Finally, Supplementary Table S4 outlines the directory structure and contents of the Zenodo repository archives.

1.1 Per-dataset baseline model performance and explanation coverage

The ConvLSTM classifier achieves a wide range of accuracies, from challenging sets like *EthanolConcentration* (32.82%) and *Fungi* (3.92%) to perfectly classified cases (*BasicMotions*, *CBF*, *Wafer*, all 100.00%). Crucially, the tables reveal a significant disparity in explanation coverage. While numeric Feature Attribution methods reliably generate outputs (SHAP succeeds in 103/103 cases, LIME in 102/103), the baseline Rule-Based method, Anchor, extracts valid rules for only 30 out of 103 evaluated datasets (∼29.1%). In contrast, our recently developed PHAR framework effectively bridges this gap: by transforming numeric attributions into robust intervals, it achieves 100% rule generation coverage (103/103) across all time series.

Table S1. Multivariate datasets: statistics, classifier accuracy, and explanation coverage.

							Feature Attribution		Rule-Based	
Time Series	Train	Test	Len.	Dim.	Classes	Acc. (%)	LIME	SHAP	Anchor	PHAR
ArticularyWordRecognition	431	144	144	9	25	97.22	✓	✓	×	✓
AtrialFibrillation	22	8	640	2	3	50.00	✓	✓	×	✓
BasicMotions	60	20	100	6	4	100.00	✓	✓	×	✓
Cricket	135	45	1197	6	12	91.11	✓	✓	×	✓
Epilepsy	206	69	206	3	4	91.30	✓	✓	×	✓
ERing	225	75	65	4	6	96.00	✓	✓	×	✓
EthanolConcentration	393	131	1751	3	4	32.82	✓	✓	✓	✓
FaceDetection	7060	2354	62	144	2	50.25	×	✓	✓	✓
FingerMovements	312	104	50	28	2	56.73	✓	✓	×	✓
HandMovementDirection	175	59	400	10	4	25.42	✓	✓	×	✓
Handwriting	750	250	152	3	26	56.40	✓	✓	×	✓
Heartbeat	306	103	405	61	2	72.82	✓	✓	×	✓
Libras	270	90	45	2	15	65.56	✓	✓	✓	✓
LSST	3693	1232	36	6	14	22.40	✓	✓	×	✓
NATOPS	270	90	51	24	6	86.67	✓	✓	×	✓
PenDigits	8244	2748	8	2	10	98.62	✓	✓	✓	✓
RacketSports	227	76	30	6	4	80.26	✓	✓	×	✓
SelfRegulationSCP1	420	141	896	6	2	90.78	✓	✓	×	✓
SelfRegulationSCP2	285	95	1152	7	2	47.37	✓	✓	×	✓
UWaveGestureLibrary	330	110	315	3	8	92.73	✓	✓	×	✓

[1] M. Mozolewski, S. Bobek, G. J. Nalepa, *"Explaining Time Series Classifiers with PHAR: Rule Extraction and Fusion from Post-hoc Attributions"*

Table S2. Univariate datasets: statistics, classifier accuracy, and explanation coverage (Part 1/2).

							Feature Attribution		Rule-Based	
Time Series	Train	Test	Len.	Dim.	Classes	Acc. (%)	LIME	SHAP	Anchor	PHAR
Adiac	585	196	176	1	37	28.06	✓	✓	×	✓
Beef	45	15	470	1	5	46.67	✓	✓	×	✓
BeetleFly	30	10	512	1	2	80.00	✓	✓	×	✓
BirdChicken	30	10	512	1	2	60.00	✓	✓	×	✓
BME	135	45	128	1	3	91.11	✓	✓	×	✓
CBF	697	233	128	1	3	100.00	✓	✓	×	✓
Chinatown	272	91	24	1	2	98.90	✓	✓	✓	✓
Coffee	42	14	286	1	2	78.57	✓	✓	×	✓
Computers	375	125	720	1	2	64.80	✓	✓	×	✓
CricketX	585	195	300	1	12	64.62	✓	✓	✓	✓
CricketY	585	195	300	1	12	62.05	✓	✓	×	✓
CricketZ	585	195	300	1	12	64.62	✓	✓	×	✓
Crop	18000	6000	46	1	24	68.68	✓	✓	×	✓
DiatomSizeReduction	241	81	345	1	4	96.30	✓	✓	×	✓
DistalPhalanxOutlineAgeGroup	404	135	80	1	3	77.78	✓	✓	✓	✓
DistalPhalanxOutlineCorrect	657	219	80	1	2	77.17	✓	✓	✓	✓
DistalPhalanxTW	404	135	80	1	6	68.89	✓	✓	×	✓
DodgerLoopDay	118	40	288	1	7	57.50	✓	✓	✓	✓
DodgerLoopGame	118	40	288	1	2	87.50	✓	✓	✓	✓
DodgerLoopWeekend	118	40	288	1	2	97.50	✓	✓	×	✓
Earthquakes	345	116	512	1	2	69.83	✓	✓	×	✓
ECG200	150	50	96	1	2	86.00	✓	✓	✓	✓
ECG5000	3750	1250	140	1	5	92.16	✓	✓	✓	✓
ECGFiveDays	663	221	136	1	2	99.55	✓	✓	✓	✓
ElectricDevices	12477	4160	96	1	7	84.69	✓	✓	×	✓
FaceFour	84	28	350	1	4	92.86	✓	✓	✓	✓
FiftyWords	678	227	270	1	47	63.00	✓	✓	×	✓
FordA	3690	1231	500	1	2	83.92	✓	✓	×	✓
FordB	3334	1112	500	1	2	85.16	✓	✓	×	✓
FreezerRegularTrain	2250	750	301	1	2	97.33	✓	✓	×	✓
FreezerSmallTrain	2158	720	301	1	2	94.17	✓	✓	×	✓
Fungi	153	51	201	1	18	3.92	✓	✓	✓	✓
GunPoint	150	50	150	1	2	84.00	✓	✓	×	✓
GunPointAgeSpan	338	113	150	1	2	90.27	✓	✓	✓	✓
GunPointMaleVersusFemale	338	113	150	1	2	100.00	✓	✓	×	✓
GunPointOldVersusYoung	338	113	150	1	2	100.00	✓	✓	×	✓
Herring	96	32	512	1	2	56.25	✓	✓	×	✓
InsectWingbeatSound	1650	550	256	1	11	65.64	✓	✓	×	✓
ItalyPowerDemand	822	274	24	1	2	95.99	✓	✓	×	✓
LargeKitchenAppliances	562	188	720	1	3	63.83	✓	✓	×	✓
Lightning2	90	31	637	1	2	54.84	✓	✓	×	✓
Lightning7	107	36	319	1	7	66.67	✓	✓	×	✓

Table S3. Univariate datasets: statistics, classifier accuracy, and explanation coverage (Part 2/2).

							Feature Attribution		Rule-Based	
Time Series	Train	Test	Len.	Dim.	Classes	Acc. (%)	LIME	SHAP	Anchor	PHAR
Meat	90	30	448	1	3	63.33	✓	✓	×	✓
MedicalImages	855	286	99	1	9	65.03	✓	✓	✓	✓
MiddlePhalanxOutlineAgeGroup	415	139	80	1	3	78.42	✓	✓	✓	✓
MiddlePhalanxOutlineCorrect	668	223	80	1	2	70.40	✓	✓	×	✓
MiddlePhalanxTW	414	139	80	1	6	64.75	✓	✓	×	✓
MoteStrain	954	318	84	1	2	95.28	✓	✓	×	✓
OliveOil	45	15	570	1	4	13.33	✓	✓	✓	✓
OSULeaf	331	111	427	1	6	60.36	✓	✓	×	✓
PhalangesOutlinesCorrect	1993	665	80	1	2	67.82	✓	✓	✓	✓
Plane	157	53	144	1	7	94.34	✓	✓	✓	✓
PowerCons	270	90	144	1	2	100.00	✓	✓	✓	✓
ProximalPhalanxOutlineAgeGroup	453	152	80	1	3	69.74	✓	✓	×	✓
ProximalPhalanxOutlineCorrect	668	223	80	1	2	71.75	✓	✓	×	✓
ProximalPhalanxTW	453	152	80	1	6	46.71	✓	✓	✓	✓
RefrigerationDevices	562	188	720	1	3	43.62	✓	✓	×	✓
ScreenType	562	188	720	1	3	40.96	✓	✓	×	✓
ShapeletSim	150	50	500	1	2	48.00	✓	✓	×	✓
ShapesAll	900	300	512	1	60	66.00	✓	✓	×	✓
SmallKitchenAppliances	562	188	720	1	3	59.57	✓	✓	×	✓
SmoothSubspace	225	75	15	1	3	93.33	✓	✓	✓	✓
SonyAIBORobotSurface1	465	156	70	1	2	98.72	✓	✓	×	✓
SonyAIBORobotSurface2	735	245	65	1	2	99.18	✓	✓	×	✓
Strawberry	737	246	235	1	2	73.58	✓	✓	✓	✓
SwedishLeaf	843	282	128	1	15	83.33	✓	✓	✓	✓
Symbols	765	255	398	1	6	94.90	✓	✓	×	✓
SyntheticControl	450	150	60	1	6	89.33	✓	✓	✓	✓
ToeSegmentation2	124	42	343	1	2	85.71	✓	✓	×	✓
Trace	150	50	275	1	4	68.00	✓	✓	✓	✓
TwoLeadECG	871	291	82	1	2	90.03	✓	✓	×	✓
TwoPatterns	3750	1250	128	1	4	99.84	✓	✓	×	✓
UMD	135	45	150	1	3	91.11	✓	✓	✓	✓
UWaveGestureLibraryAll	3358	1120	945	1	8	95.54	✓	✓	×	✓
UWaveGestureLibraryX	3358	1120	315	1	8	80.89	✓	✓	×	✓
UWaveGestureLibraryY	3358	1120	315	1	8	71.96	✓	✓	×	✓
UWaveGestureLibraryZ	3358	1120	315	1	8	75.62	✓	✓	×	✓
Wafer	5373	1791	152	1	2	100.00	✓	✓	✓	✓
Wine	83	28	234	1	2	60.71	✓	✓	×	✓
WordSynonyms	678	227	270	1	25	67.84	✓	✓	×	✓
Worms	193	65	900	1	5	52.31	✓	✓	×	✓
WormsTwoClass	193	65	900	1	2	50.77	✓	✓	×	✓
Yoga	2475	825	426	1	2	90.91	✓	✓	×	✓

1.2 Repository archives and internal contents

All filenames in the Zenodo record follow the pattern `{uni|multi}_{series_name}_*`, which keeps univariate and multivariate variants distinct. File-level conventions, such as the use of `.pickle` for NumPy arrays and Python objects, alongside `.json` and `.jsonl` for hyperparameter optimization metadata for PHAR method, are shared across datasets to facilitate loading, visualising, and cross-comparing explanation values. Supplementary Table S4 summarises the repository structure.

Table S4. Overview of repository files and their contents

Archive Name	Description and Internal Contents
`train_test.zip`	Contains per-dataset archives named `{uni|multi}_{name}_train_and_test.zip`. Each inner archive holds four NumPy arrays in `.pickle` format: `trainX.pickle`, `trainy.pickle`, `testX.pickle`, `testy.pickle`, corresponding to the standardised 75/25 train/test split.
`models.zip`	Contains TensorFlow SavedModel directories, one per dataset, named `{uni|multi}_{series_name}_model`. Each directory stores the pretrained ConvLSTM-based model weights and model configuration.
`shap.zip`	Contains SHAP explanation values for each dataset in archives named `{series_name}_shap_values.zip`. Inside each archive are two NumPy arrays in `.pickle` format: `svtr.pickle` (training-set SHAP values) and `svts.pickle` (test-set SHAP values).
`lime.zip`	Contains LIME explanation values for each dataset in archives named `{series_name}_lime_values.zip`. Each archive holds two NumPy arrays in `.pickle` format: `lvtr.pickle` (training-set LIME values) and `lvts.pickle` (test-set LIME values).
`anchor.zip`	Contains Anchor rule-based explanations for each dataset in archives named `{series_name}_anchor_values.zip`. Each inner archive includes two Python pickles in list-of-lists format: `avtr.pickle` (training-set Anchor rules) and `avts.pickle` (test-set Anchor rules).
`phar.zip`	Contains PHAR rule-based explanations and hyperparameter optimization logs for each dataset in archives named `{series_name}_phar_values.zip`. Each inner archive includes two Python pickles (`pvtr.pickle` and `pvts.pickle`) following the Anchor list-of-lists format, alongside two structured metadata files detailing the optimization process:<br>• **`phar_metadata.json`**: Captures the optimal configuration found. Key objects include `params` (e.g., base explainer, threshold percentile, perturb sigma), `dynamic_params` (e.g., perturbation samples count), `metrics` (aggregated statistics for confidence, coverage, and sparsity), a `rules` array detailing the rules extracted specifically for the optimization sample pool, and an `artifacts_generated` list pointing to the final serialized outputs.<br>• **`phar_trials_log.jsonl`**: A JSON Lines file recording the complete history of all optimization trials. Each line details a single trial's configuration, its specific evaluated `metrics`, `total_time`, and the intermediate `rules` generated for the sample pool during that step.
